# Supplementary material for: High-throughput sequencing defines donor and recipient HLA B-cell epitope frequencies for prospective matching in transplantation
Source: Commun Biol. 2021 May 14;4:583. doi: 10.1038/s42003-021-01989-3 (PMC8121953; doi:10.1038/s42003-021-01989-3)
Supplement: Supplementary file 2 — Supplementary Information [file 42003_2021_1989_MOESM2_ESM.pdf]

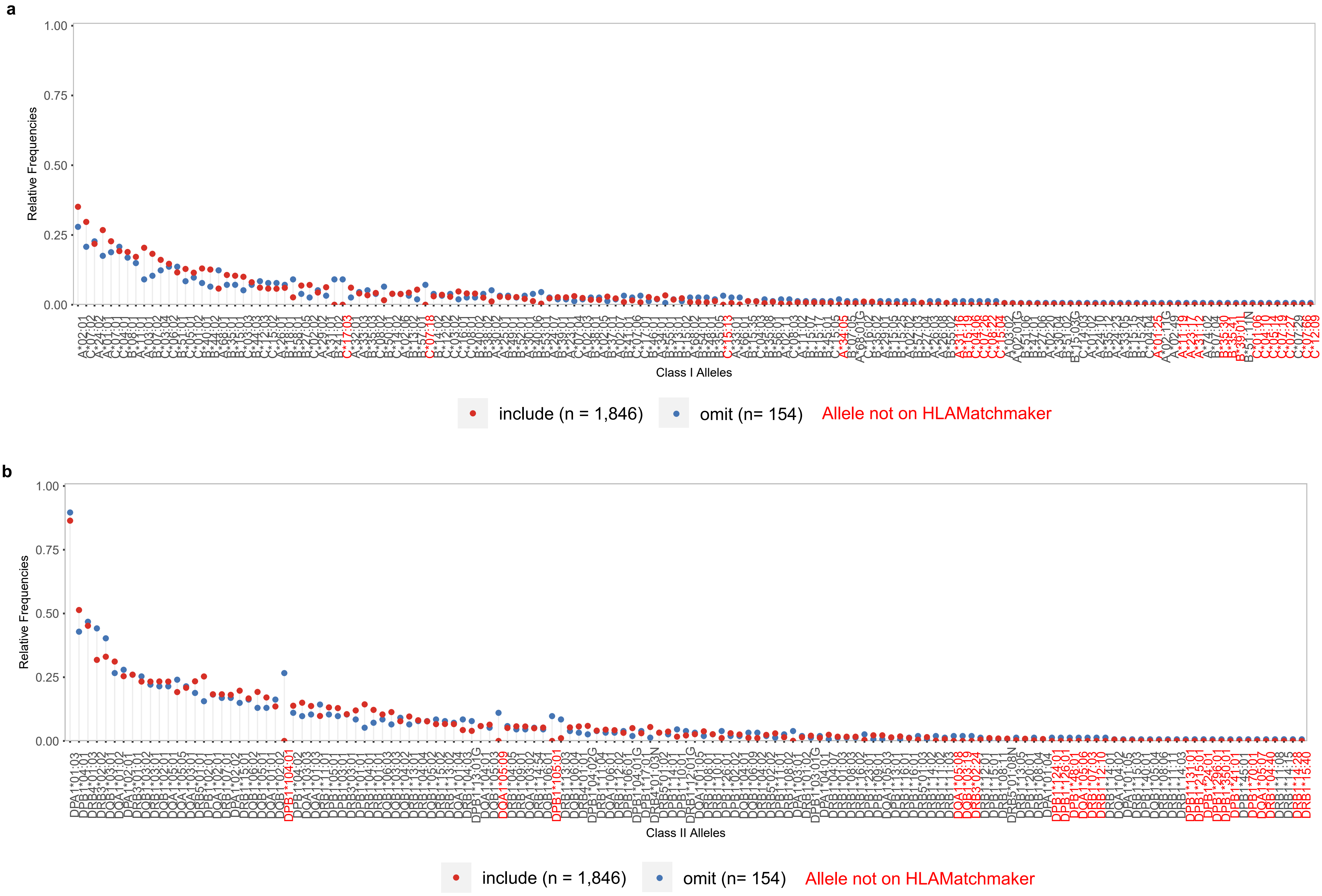


**Supplemental Figure 1. A comparison of the relative frequencies in observed alleles present in the omitted and included subjects in the study population.** Relative frequencies were calculated as the proportion of subjects expressing a particular alleles within the study cohort (*include*, n = 1846) and omitted subjects (*omit*, n = 154). (a) and (b) depict the allele frequencies by class I and class II, respectively.

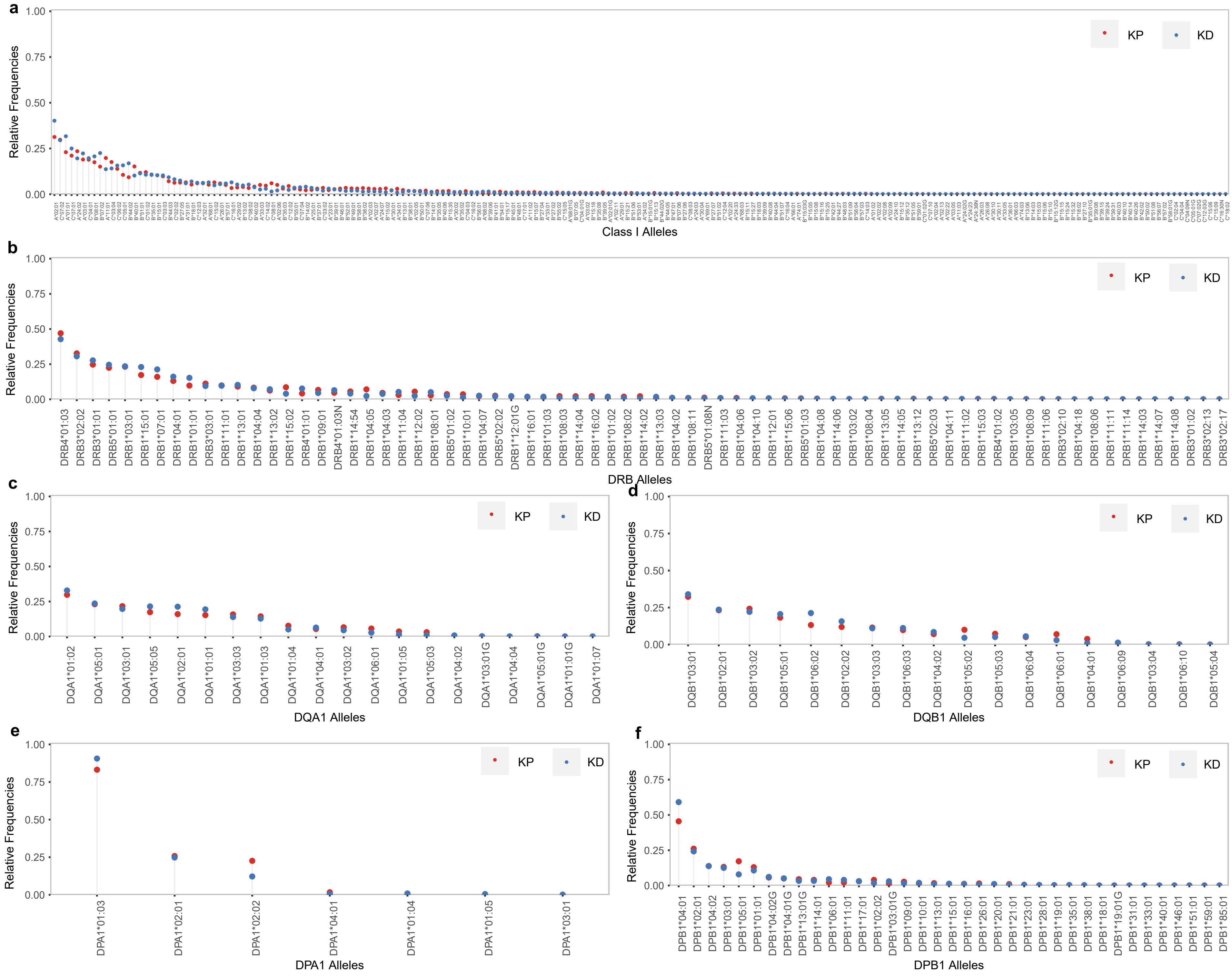


**Supplemental Figure 2. The relative frequencies of the HLA alleles in the study population categorized by class I genes and individual class II genes.** Relative frequencies were calculated as the proportion of subjects of subjects expressing a particular allele within kidney patients (n=1049) or donors (n=797). (a) class I, (b) DRB1/3/4/5, (c) DQA1, (d) DQB1, (e) DPA1, and (f) DPB1 observed alleles. *KP = Kidney patients, KD = kidney donors.*

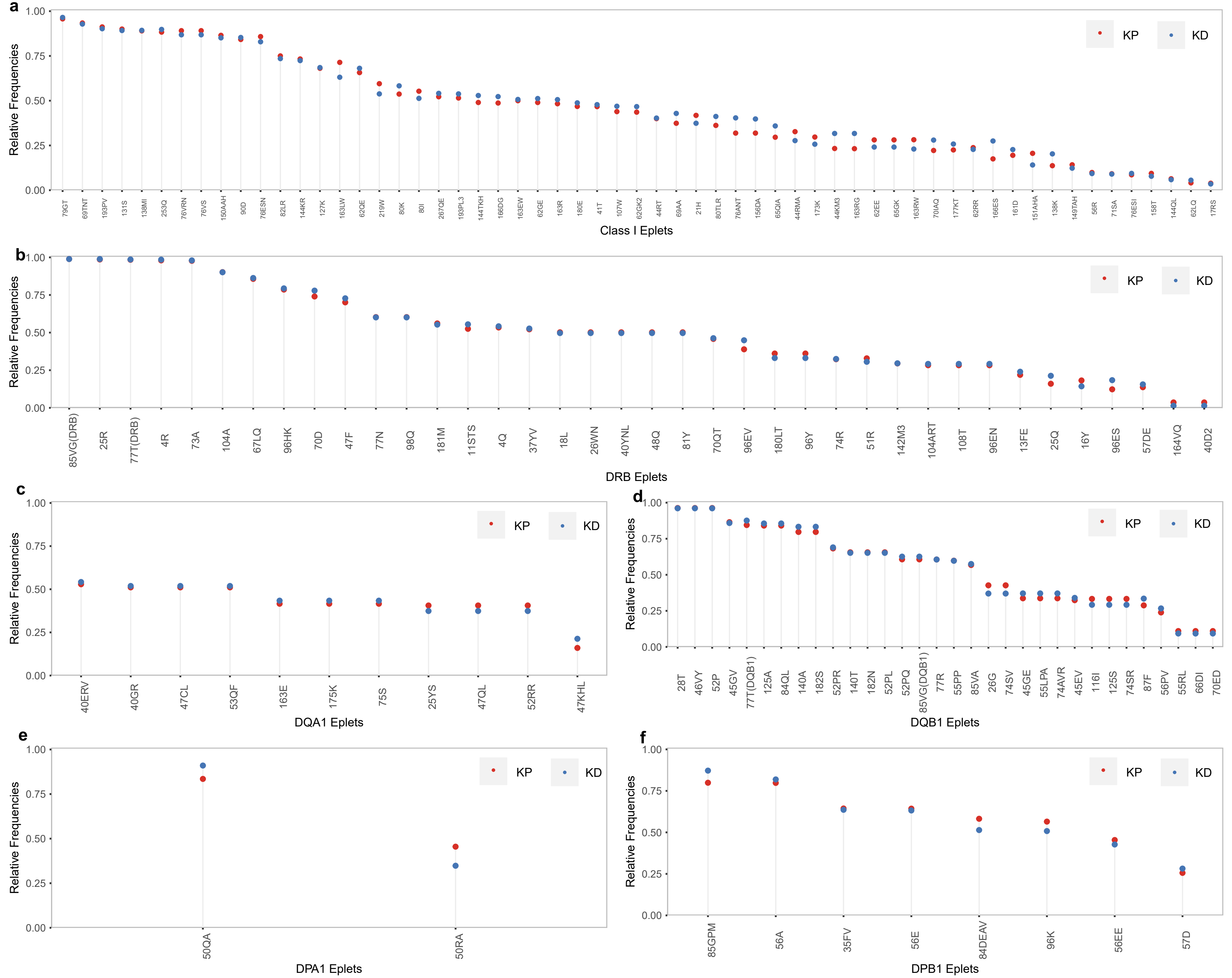


**Supplemental Figure 3. The relative frequencies of the HLA eplets in the study population categorized by class I genes and individual class II genes.** Relative frequencies were calculated as the proportion of subjects of subjects expressing a particular eplet within kidney patients (n=1049) or donors (n=797). (a) class I, (b) DRB1/3/4/5, (c) DQA1, (d) DQB1, (e) DPA1, and (f) DPB1 observed alleles. *KP = Kidney patients, KD = kidney donors.*

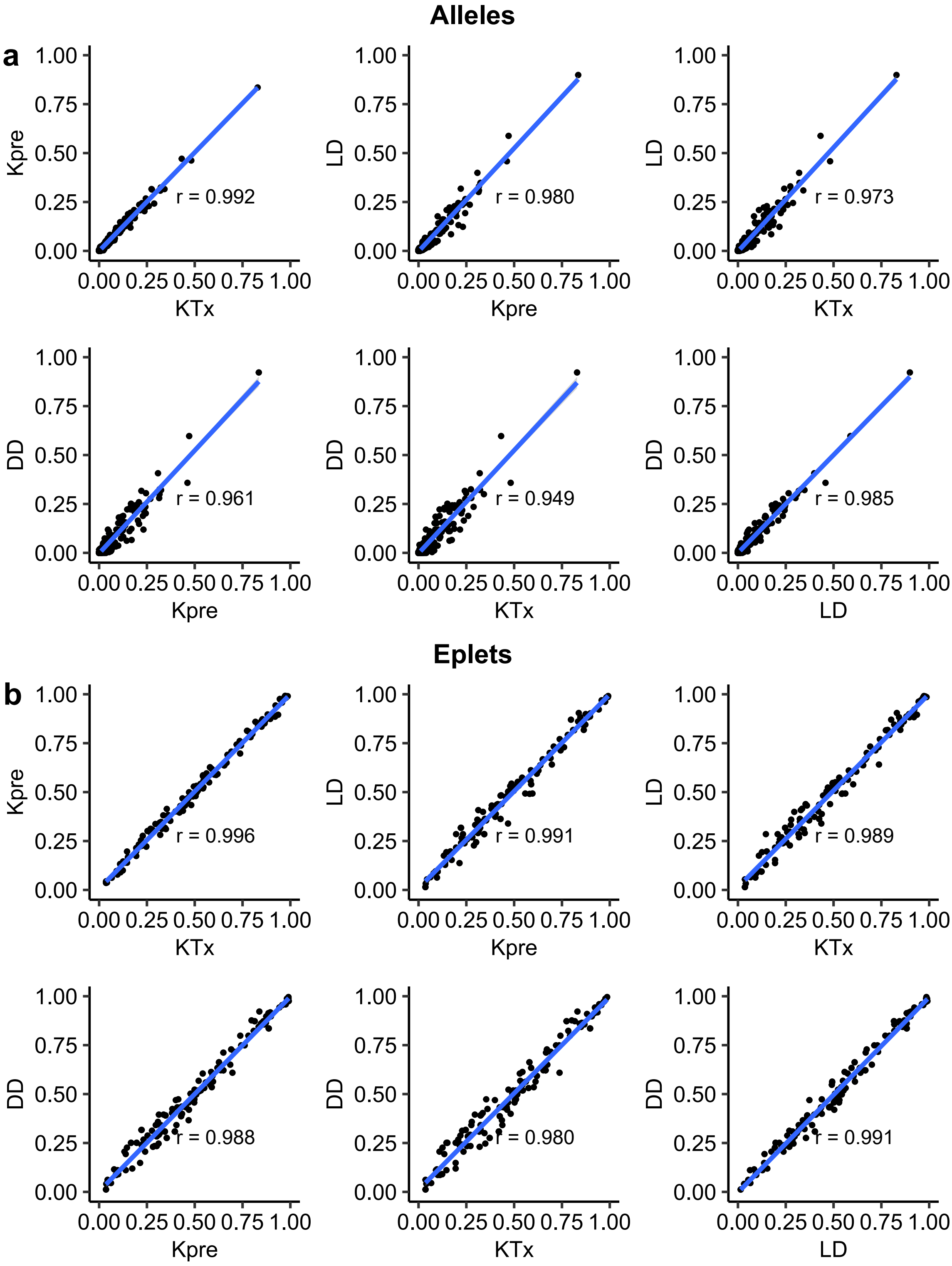


**Supplemental Figure 4. Pairwise analysis between patient and donor subgroups of allele and eplet frequencies.** Frequencies were calculated as the patients and donors expressing a particular allele or eplet. The line and correlation coeffiecient were calculated using Pearson Correlation. (a) Each dot represents an allele  and its frequency for patients (n=1049) is plotted on the x axis and its frequency for donors (n=797) on the y axis. (b) As above but for eplets. Kpre = pre-transplant recipients, Ktx = post-transplant recipients, LD = potentia lliving donors, and DD = deceased donors.

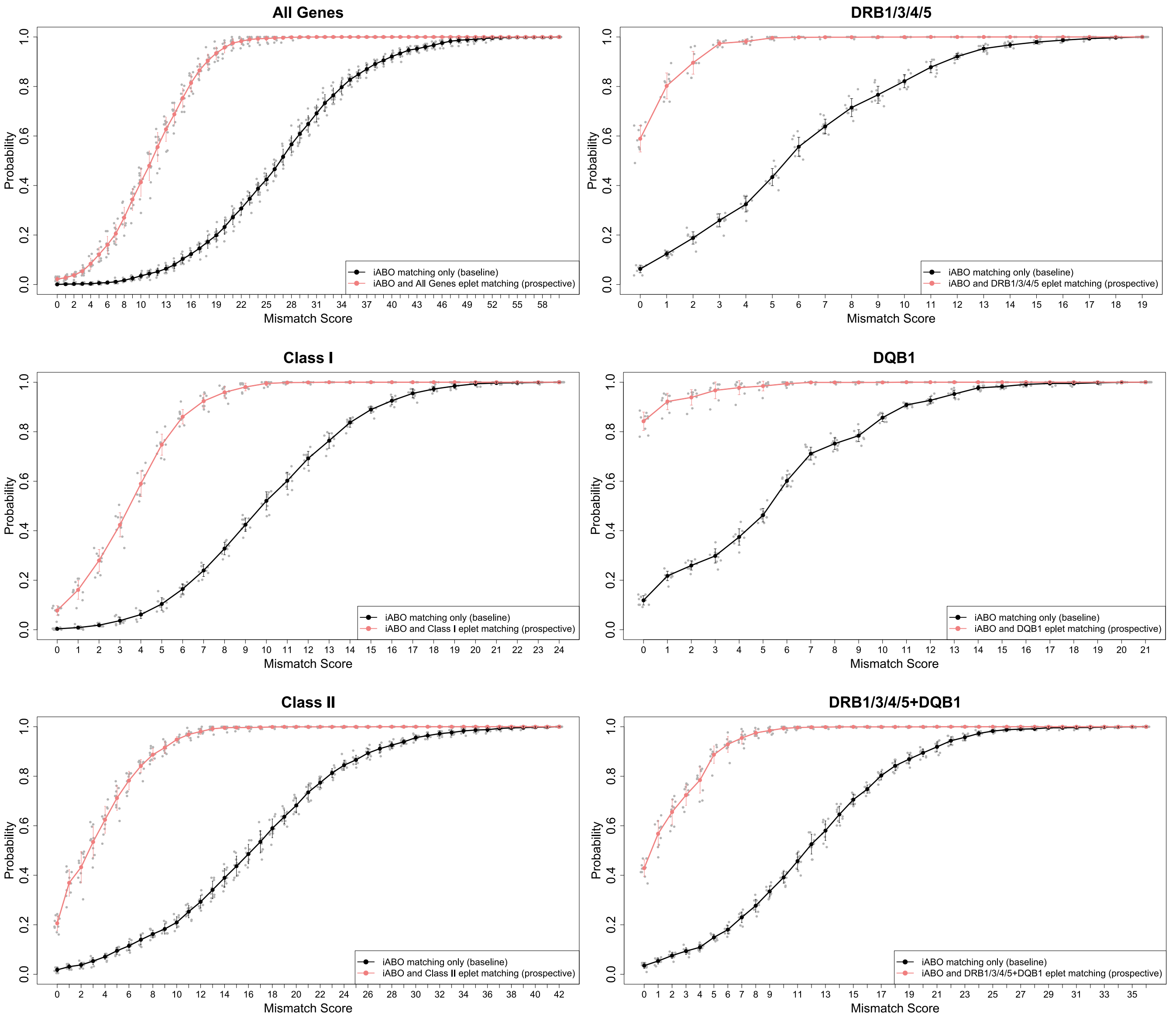


**Supplemental Figure 5. Cumulative probabilities of eplet mismatches in the average province matching simulations with and without prospective eplet matching.** Matching simulations incorporated prospective eplet and blood group matching (red curve) and baseline blood group matching only (black curve) in kidney patients and deceased donors. Plots represent simulations using an averaged province Canadian waitlist (n=290) and an annual deceased donor rate (n=109) according to the Canadian Organ Replacement Register. Simulations were performed across all HLA genes, class I genes, class II genes, DRB1/3/4/5, DQB1, and DRB1/3/4/5+DQB1 combined. Eplet mismatch scores for the respective genes on the x axis are plotted against the averaged cumulative probability of these scores in the matched population. Jittered grey dots represent the cumulative probabilities at each individual simulation. Error bars are calculated as the standard deviation ten repeated independent simulation runs.

**Supplemental Table 1. List of observed alleles and their relative frequencies in the total population that are absent from HLAMatchmaker class I v02 and class II v2.2.**

| **HLA-A** | | **HLA-C** | | **HLA-DPB1** | | **HLA-DQA1** | |
|---|---|---|---|---|---|---|---|
| A*01:25 | 0.0005 | C*01:06 | 0.0005 | DPB1*104:01 | 0.0205 | DQA1*05:06 | 0.001 |
| A*11:19 | 0.0005 | C*04:06 | 0.001 | DPB1*105:01 | 0.0075 | DQA1*05:07 | 0.0005 |
| A*23:17 | 0.0005 | C*04:10 | 0.0005 | DPB1*124:01 | 0.001 | DQA1*05:08 | 0.0015 |
| A*31:12 | 0.0005 | C*05:14 | 0.0005 | DPB1*126:01 | 0.001 | DQA1*05:09 | 0.0085 |
| A*31:16 | 0.001 | C*07:18 | 0.0055 | DPB1*131:01 | 0.0005 | **HLA-DQB1** | |
| A*34:05 | 0.0015 | C*07:19 | 0.0005 | DPB1*215:01 | 0.0005 | DQB1*03:19 | 0.0015 |
| **HLA-B** | | C*07:26 | 0.001 | DPB1*24:01 | 0.001 | **HLA-DRB1** | |
| B*15:19 | 0.001 | C*07:27 | 0.0005 | DPB1*296:01 | 0.0005 | DRB1*04:40 | 0.0005 |
| B*35:30 | 0.0005 | C*07:66 | 0.0005 | DPB1*350:01 | 0.0005 | DRB1*07:03 | 0.001 |
| B*35:41 | 0.0005 | C*08:22 | 0.001 | DPB1*41:01 | 0.0005 | DRB1*12:10 | 0.001 |
| B*39:01L | 0.0005 | C*12:09 | 0.0005 | DPB1*48:01 | 0.001 | DRB1*14:28 | 0.0005 |
| | | C*15:04 | 0.001 | DPB1*70:01 | 0.0005 | DRB1*15:40 | 0.0005 |
| | | C*15:13 | 0.0025 | | | **HLA-DRB3** | |
| | | C*17:03 | 0.007 | | | DRB3*02:24 | 0.0015 |

**Supplemental Table 2. List of interlocus eplets and their relative frequencies.**

| | | Relative Frequency | | |
|---|---|---|---|---|
| **Eplet** | **Gene** | **Kidney Patients (KP)** | **Kidney Donors (KD)** | **Total Population** |
| 156DA | B,C | 0.319 | 0.398 | 0.353 |
| 163EW | A,B,C | 0.500 | 0.507 | 0.503 |
| 163LW | B,C | 0.714 | 0.631 | 0.678 |
| 166DG | A,B | 0.487 | 0.523 | 0.503 |
| 193PV | B,C | 0.912 | 0.902 | 0.908 |
| 253Q | A,C | 0.883 | 0.898 | 0.889 |
| 62GE | A,B | 0.490 | 0.512 | 0.499 |
| 62RR | A,B | 0.238 | 0.228 | 0.234 |
| 76VRN | B,C | 0.891 | 0.868 | 0.881 |
| 76VS | B,C | 0.891 | 0.868 | 0.881 |
| 80I | A,B | 0.553 | 0.513 | 0.536 |
| 82LR | A,B | 0.750 | 0.735 | 0.744 |
| 90D | A,C | 0.842 | 0.853 | 0.847 |
| 104A | DRB1,3,5 | 0.902 | 0.901 | 0.901 |
| 181M | DRB1,4 | 0.561 | 0.553 | 0.557 |
| 25R | DRB1,3,5 | 0.986 | 0.989 | 0.987 |
| 4Q | DRB1,4 | 0.534 | 0.542 | 0.537 |
| 4R | DRB1,3,5 | 0.980 | 0.985 | 0.982 |
| 67LQ | DRB1,3 | 0.857 | 0.863 | 0.860 |
| 70D | DRB1,5 | 0.740 | 0.779 | 0.757 |
| 73A | DRB1,4,5 | 0.977 | 0.980 | 0.978 |
| 74R | DRB1,3 | 0.323 | 0.325 | 0.324 |
| 77N | DRB1,3 | 0.603 | 0.601 | 0.602 |
| 77T(DRB) | DRB1,4,5 | 0.845 | 0.876 | 0.985 |
| 85VG(DRB) | DRB1,3,5 | 0.607 | 0.626 | 0.989 |
| 96EV | DRB1,5 | 0.389 | 0.449 | 0.415 |
| 96HK | DRB1,3 | 0.786 | 0.794 | 0.789 |
