# Supplementary material for: High-throughput sequencing defines donor and recipient HLA B-cell epitope frequencies for prospective matching in transplantation
Source: Commun Biol. 2021 May 14;4:583. doi: 10.1038/s42003-021-01989-3 (PMC8121953; doi:10.1038/s42003-021-01989-3)
Supplement: Supplementary file 6 — Description of Additional Supplementary Files [file 42003_2021_1989_MOESM6_ESM.pdf]

Description of Additional Supplementary Files

File name: Supplemental Data 1

Description: The observed alleles in the study population and their relative frequencies.

File name: Supplemental Data 2

Description: The observed eplets in the study population and their relative frequencies.

File name: Supplemental Data 3

Description: Cumulative eplet mismatch scores in the prospective eplet matching simulations.

Scores are averaged over a set of 10 repeated simulations.
